# Compact-Morphology-based poly-metallic Nodule Delineation

**DOI:** 10.1038/s41598-017-13335-x

**Published:** 2017-10-17

**Authors:** Timm Schoening, Daniel O. B. Jones, Jens Greinert

**Affiliations:** 1 GEOMAR Helmholtz Centre for Ocean Research, Kiel, Germany; 2 National Oceanography Centre, Southampton, UK

## Abstract

Poly-metallic nodules are a marine resource considered for deep sea mining. Assessing nodule abundance is of interest for mining companies and to monitor potential environmental impact. Optical seafloor imaging allows quantifying poly-metallic nodule abundance at spatial scales from centimetres to square kilometres. Towed cameras and diving robots acquire high-resolution imagery that allow detecting individual nodules and measure their sizes. Spatial abundance statistics can be computed from these size measurements, providing e.g. seafloor coverage in percent and the nodule size distribution. Detecting nodules requires segmentation of nodule pixels from pixels showing sediment background. Semi-supervised pattern recognition has been proposed to automate this task. Existing nodule segmentation algorithms employ machine learning that trains a classifier to segment the nodules in a high-dimensional feature space. Here, a rapid nodule segmentation algorithm is presented. It omits computation-intense feature-based classification and employs image processing only. It exploits a nodule compactness heuristic to delineate individual nodules. Complex machine learning methods are avoided to keep the algorithm simple and fast. The algorithm has successfully been applied to different image datasets. These data sets were acquired by different cameras, camera platforms and in varying illumination conditions. Their successful analysis shows the broad applicability of the proposed method.

## Introduction

Poly-metallic nodules (PMNs - also referred to as manganese nodules) are a marine mineral resource. They contain relevant concentrations of Copper, Nickel and Cobalt^1^. Most PMN research and resource assessment focusses on the deep abyssal plains in the eastern Pacific Ocean. Economically interesting areas were found within the *Clarion Clipperton Zone* (CCZ) where several countries are exploring PMN claims. These are license areas provided to the countries through the International Seabed Authority.

International research projects are investigating aspects of nodule mining (*Ecological Aspects of Deep-Sea Mining*: https://jpio-miningimpact.geomar.de; *Managing Impacts of Deep-Sea Resource Exploitation*: https://www.eu-midas.net/). Current foci of these research projects lie on environmental aspects: e.g. epifauna associated with PMNs, mining-induced habitat disturbances and the resilience of the ecosystem to cope with this anthropogenic pressure^2,3^.

Quantitative measurements of PMN abundance are required for various applications. Individual nodule sizes (in cm^2^) provide basic measurements that can be aggregated to create descriptive spatial statistics. Examples are PMN density (nodules/m^2^) and seafloor coverage (in %) that data can related to environmental parameters, like faunal densities or chemical gradients.

Quantitative nodule information is traditionally provided by two methods. First, by physical sea floor sampling, e.g. using *box cores*. Second, by hydro-acoustic sensors, e.g. *Multi-beam Echo Sounder* (MBES) or *Side Scan Sonar* (SSS). Physical sampling by box cores provides precise size measurements of individual PMNs at minimum areal coverage. MBES and SSS provide high areal coverage but minimum quantitative resolution.

It is possible to correlate hydro-acoustic data and physical sampling for specific PMN abundances, but nodules need to be frequent and homogeneously distributed. Box cores can provide misleading data in heterogeneously populated areas and might even provide empty measurements despite an actual PMN occurrence in sparsely populated areas.

**Figure 1:**
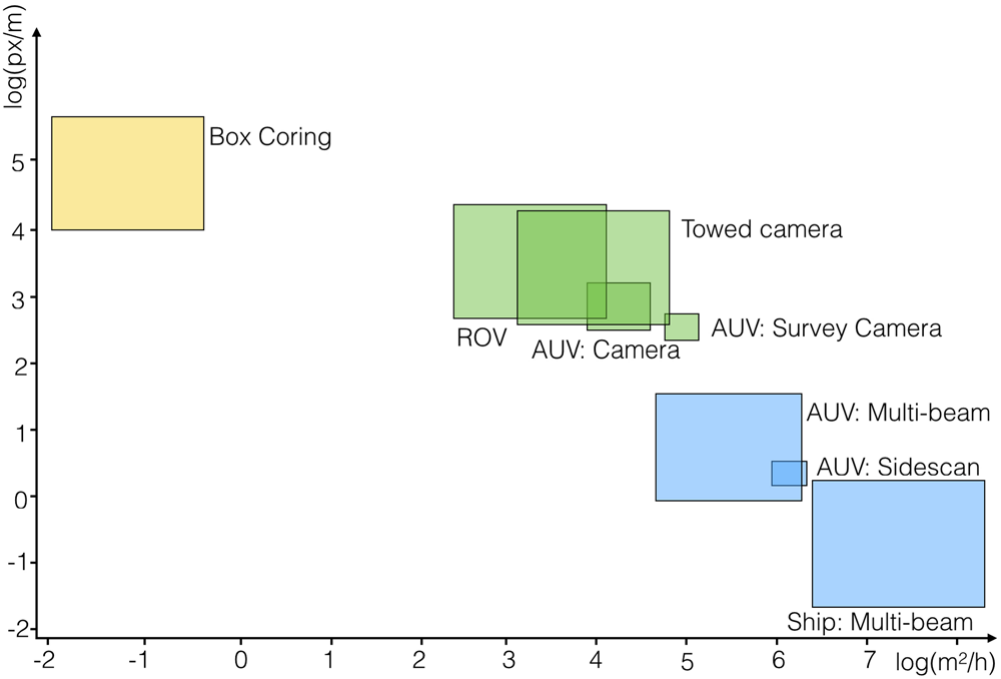
Comparison of mapping and sampling technology by their resolution (in [log _10_(px/m)]) and aerial coverage (in [log _10_(m^2^/h)]). Physical sampling with box cores (yellow box) provides maximal resolution at minimal aerial coverage while hydro-acoustic (Multi-beam Echo Sounder (MBES) and Side Scan Sonar (SSS) - blue boxes) methods provide minimum resolution at maximum aerial coverage. Optical imaging (green boxes) provides a bridge technology that enables seafloor measurements at intermediate resolution and aerial coverage. Hydro-acoustic and optical methods are separated by platform (Ship, AUV, ROV, etc.) and sensor (MBES, SSS, Camera (e.g. Survey Camera^5^), etc.).

Optical imaging provides a bridge technology between these two methods. It features higher quantitative resolution than hydro-acoustics and higher areal coverage than physical sampling (see Fig. 1)^4^. It can be used to measure local PMN heterogeneity as well as regional abundance.

Semantics have to be assigned to pixels to determine nodule quantities in optical imagery. The objects of interest, i.e. PMNs, have to be segmented from the sediment background making this semantic segmentation is a binary task. Each pixel of an image needs to be assigned to either the PMN class ${\omega }_{0}$ or the sediment background class ${\omega }_{1}$.

After the segmentation, individual PMNs need to be delineated. This delineation provides nodule sizes in cm^2^ - like physical sampling. By aggregating those size measurements imaging provides spatial nodule abundance statistics for large seafloor areas - like hydro-acoustic mapping. Examples of spatial statistics are size distributions, seafloor percent coverage and the number of nodules per area.

Traditionally, manual image annotation is conducted to add semantic information to images and specific tools for manual annotation of underwater imagery have been developed (e.g. *Squidle* or *BIIGLE* ^6,7^). In manual annotation, human experts inspect images and perform two steps: detection and classification of objects of interest. Detections are quantified by geometrical markers placed on top of the images (e.g. rectangles, polygons). Classifications are quantified by category names (e.g. “nodule”).

Classification is easy for the binary nodule case (${\omega }_{0}$ or ${\omega }_{1}$), detection however is complicated. Nodules are embedded within the sediment and a gradual transition is frequent between the two classes. Delineating nodules manually can not be achieved in a pixel-perfect way and early methods use percent coverage to measure nodule abundance in images^8,9^. These coverage measurements have to be seen as subjective *inspections* rather than objective *annotations*. Also, all manual image interpretation - inspection and annotation - is prone to bias by human factors like fatigue. Several studies showed that manual underwater image annotation is an error-prone task^10–12^ as it is in other image analysis domains^13^.

Pattern Recognition (PR) has been proposed to reduce human observer bias. PR-based image analysis methods are usually developed for imagery acquired in air. Their application to in-water imagery is complicated by the underwater environment due to physical factors like scattering, wavelength-dependent light absorption and inhomogeneous illumination. Biological factors add turbidity, marine snow and biofouling of image acquisition gear as further challenges. Some underwater imagery can be pre-processed to make in-air PR methods applicable^14^.

Successful underwater application of PR has been performed for specific use cases. Fish were classified by a deep neural network^15^ and by manually tuning shape features^16^. Nematode biomass was assessed by manually tuning intensity thresholds^17^ and scallop abundance has been quantified using Adaboost^18^. General-purpose megafauna detection has been initiated using rich feature representations and Support Vector Machines^11^.

Efforts to automate the PMN segmentation have emerged as well but because of the aforementioned challenges of underwater imaging, traditional segmentation methods like *Region growing* ^19^, Pyramid linking^20^, Normalized-cuts^21^, Mean-shift^22^ and Otsu thresholding are not directly applicable^23^. Otsu thresholding for example heuristically tunes a threshold value based on the colour frequencies in an image but does not take the spatial distribution of colours into account.

Successful PMN segmentation methods are characterised by varying manual annotation efforts and intense computational runtimes, which is higher than the image acquisition time. An early approach to assess PMNs provides seafloor percent coverage only^9^. This approach requires few manual annotations as input for an unsupervised pixel clustering. A more sophisticated method implements manual annotation of clusters in a feature space^24^. Parts of this ‘pixel clustering’ method have been sped up using high-performance computing techniques^25^. Other parts of this method were fully automated at increased computational cost based on a compactness criterion^23^.

All methods mentioned above were tuned for specific image data sets. Those data sets are characterised by a particular illumination pattern, prevalence of backscatter and camera view angle. One method was successfully linked to hydro-acoustic measurements and contributed to PMN abundance assessment on a spatial scale larger than 1,000 m^2^ ^26^.

Here, the *Compact Morphology-based Nodule Delineation* (CoMoNoD) method is presented. It combines the advantages of the above-mentioned methods but overcomes some of their individual shortcomings. *CoMoNoD* competes by neglecting time-consuming feature computation and machine learning. It requires no manual image annotation, applies data-driven parameter tuning and is computationally optimised. *CoMoNoD* maximises the between-class contrast by a compactness criterion, similar to the criterion proposed in^23^. Comparable to the Otsu method, a colour threshold is determined. In *CoMoNoD* the threshold is based on spatial colour distributions within the image. The algorithm is implemented in a GPU-optimised way to allow for rapid image processing. Images acquired at 1 Hz can be processed in realtime on one computer. This provides PMN abundance assessments shortly after image acquisition and thus helps scientists to e.g. pin-pointing subsequent sampling locations during research cruises based on the determined spatial nodule statistics.

## Material

**Figure 2:**
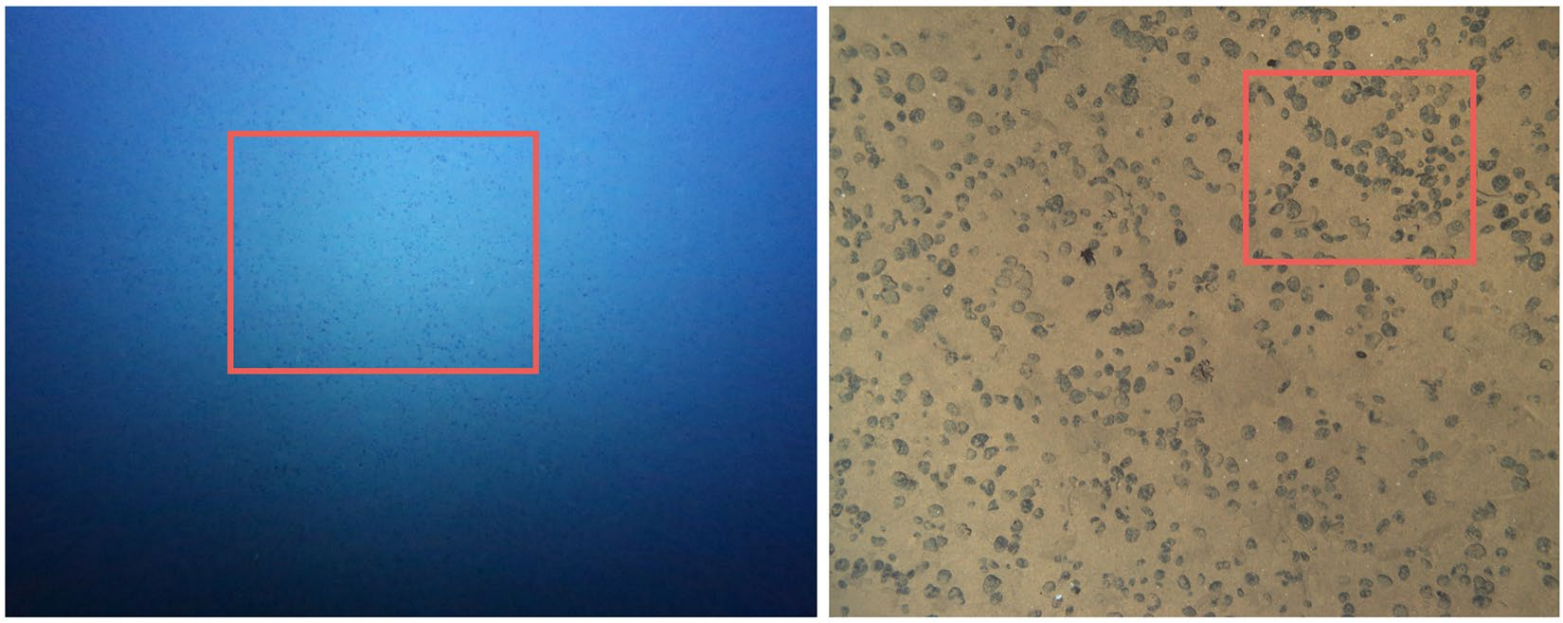
Two example images from ${I}^{\mathrm{(1)}}$ (left) and ${I}^{\mathrm{(2)}}$ (right). Both were acquired by an autonomous underwater vehicle (AUV). The left image was acquired by the *Deep Survey Camera* on board the GEOMAR AUV *Abyss* ^5^. *Abyss* flew in an altitude of ca. 8.9 m above the seafloor. Image in ${I}^{\mathrm{(1)}}$ on average show a seafloor area of ca. 180 m^2^. The image on the right was acquired by NOCS’ AUV *Autosub6000*. It flew in an altitude of ca. 2.6 m. Images in ${I}^{\mathrm{(2)}}$ show a seafloor area of ca. 1.4 m^2^. Red boxes mark sections that are shown as zoom-ins in following figures.

*CoMoNoD* was applied to two diverse image sets, ${I}^{\mathrm{(1)}}$ and ${I}^{\mathrm{(2)}}$, showing poly-metallic nodules. Both image sets were acquired in the deep sea of the Pacific Ocean. The images show a vertical view down onto the seafloor (see Fig. 2).

Image set ${I}^{\mathrm{(1)}}$ ($|{I}^{\mathrm{(1)}}|=\mathrm{34,200}$) was acquired by GEOMAR using the *Deep Survey Camera* ^5^ on board AUV *Abyss*. Images were acquired in the DISCOL experimental area of the Peru Basin (station SO242-1_083_AUV10^27^). The imaged area was chosen to reinvestigate a benthic disturbance experiment conducted in 1989^28^. ${I}^{\mathrm{(1)}}$ is a subset of a much larger AUV image collection acquired during the *Ecological Impacts of Deep Sea Mining* cruises SO239^29^ and SO242-1^27^. Image and meta data are available through OSIS (https://portal.geomar.de) and the web-based image annotation software DIAS (https://dias.geomar.de). The images in ${I}^{\mathrm{(1)}}$ were acquired from an average altitude of 7.5 m above the sea floor. A FishEye lens was used to capture a 90° field of view. FishEye un-distortion was applied to create rectified images of 4096 (3072) pixel width (height). Resolution in the rectified images is ca. 1 px/cm. Those rectified images were then analysed with *CoMoNoD*.

Image set ${I}^{\mathrm{(2)}}$ ($|{I}^{\mathrm{(2)}}|=\mathrm{88,630}$) was acquired by the National Oceanography Centre Southampton (NOCS) in an *Area of Particular Environmental Interest* (APEI No. 6) in the CCZ, using AUV *Autosub6000* ^30^. The images in ${I}^{\mathrm{(2)}}$ were acquired from an average altitude of 3 m. They feature a footprint of ca. 1.8 m^2^ and a resolution of ca. 16 px/cm.

The segmentation of nodules and sediment is considered a binary task for ${I}^{\mathrm{(1)}}$ and ${I}^{\mathrm{(2)}}$. As less than 3% of the image pixels show other objects like fauna, these objects are neglected in the analysis. Both image sets come with meta data for each image including latitude and longitude for geo-referencing as well as altitude to compute image footprints in m^2^.

## Method

**Figure 3:**
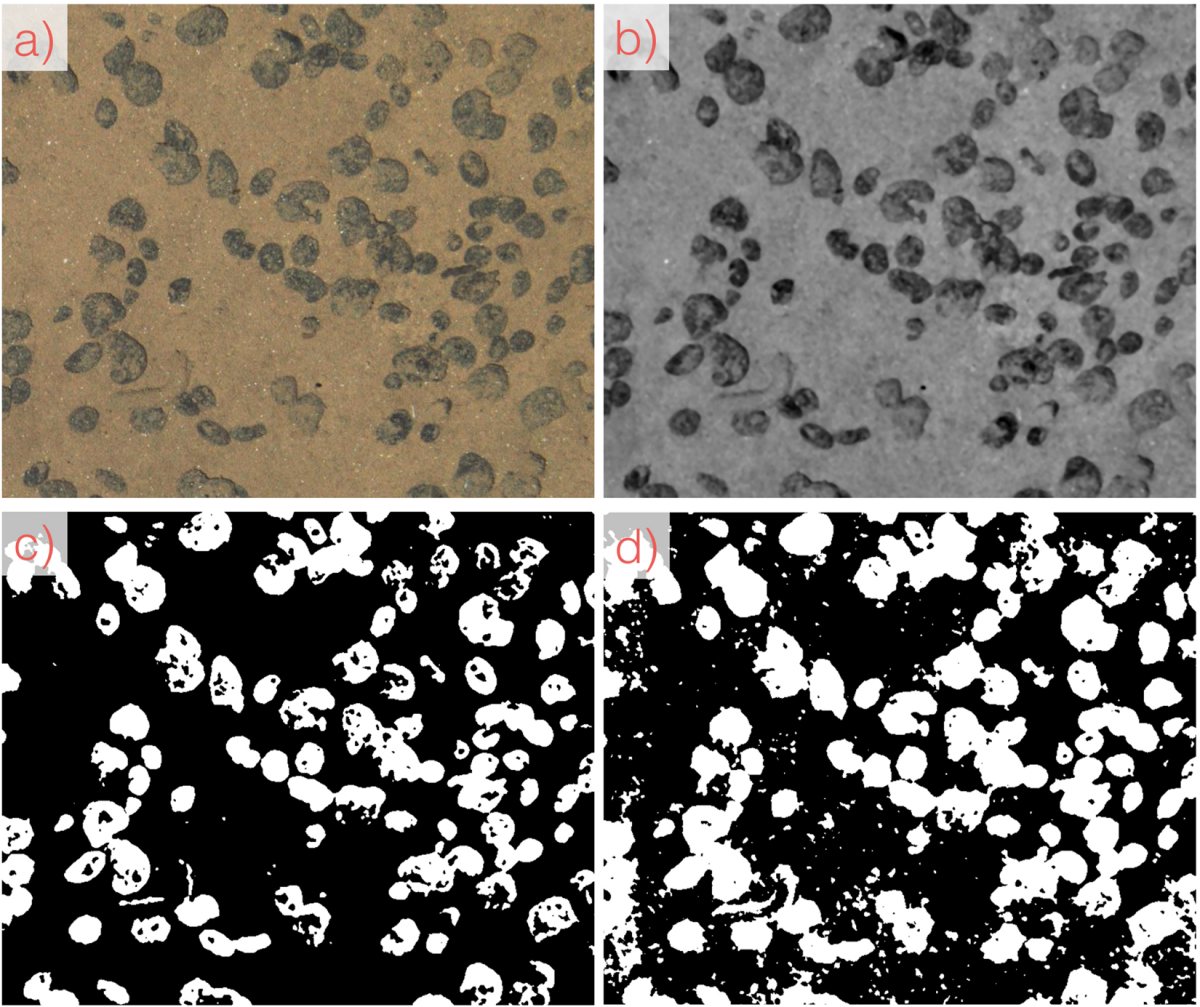
Workflow of image processing steps for contrast maximisation in the first phase of *CoMoNoD*. A zoom-in of the entire image is shown (see Fig. 2). (**a**) Shows the input image from ${I}^{\mathrm{(2)}}$ (ca. 1/10th of source image). (**b**) Shows the colour corrected version after applying the *fSpice* method. (**c**) Shows the contrast-maximised binary image ${B}_{i}$ after applying the heuristically tuned threshold ${t}_{i}$. (**d**) Shows a noisy result created by a threshold ${t}_{i}$ that was chosen too large (by erroneously setting ${\theta }_{\gamma }$ too high).

**Figure 4:**
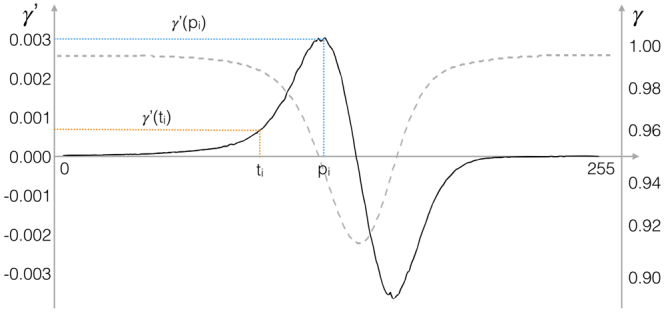
Plot of the compactness curve $\gamma (\cdot )$ (dashed, grey curve) and the first derivate $\gamma ^{\prime} (\cdot )$ (solid, black curve). The curves represent the compactness in one image from ${I}^{\mathrm{(1)}}$. To select the intensity threshold ${t}_{i}$, first the peak ${p}_{i}$ is determined in $\gamma ^{\prime} (\cdot )$. Then, ${t}_{i}$ is chosen as the highest value $b < {p}_{i}$ for which $\gamma ^{\prime} (b) < \gamma ^{\prime} ({p}_{i})\ast {\theta }_{\gamma }$.

**Figure 5:**
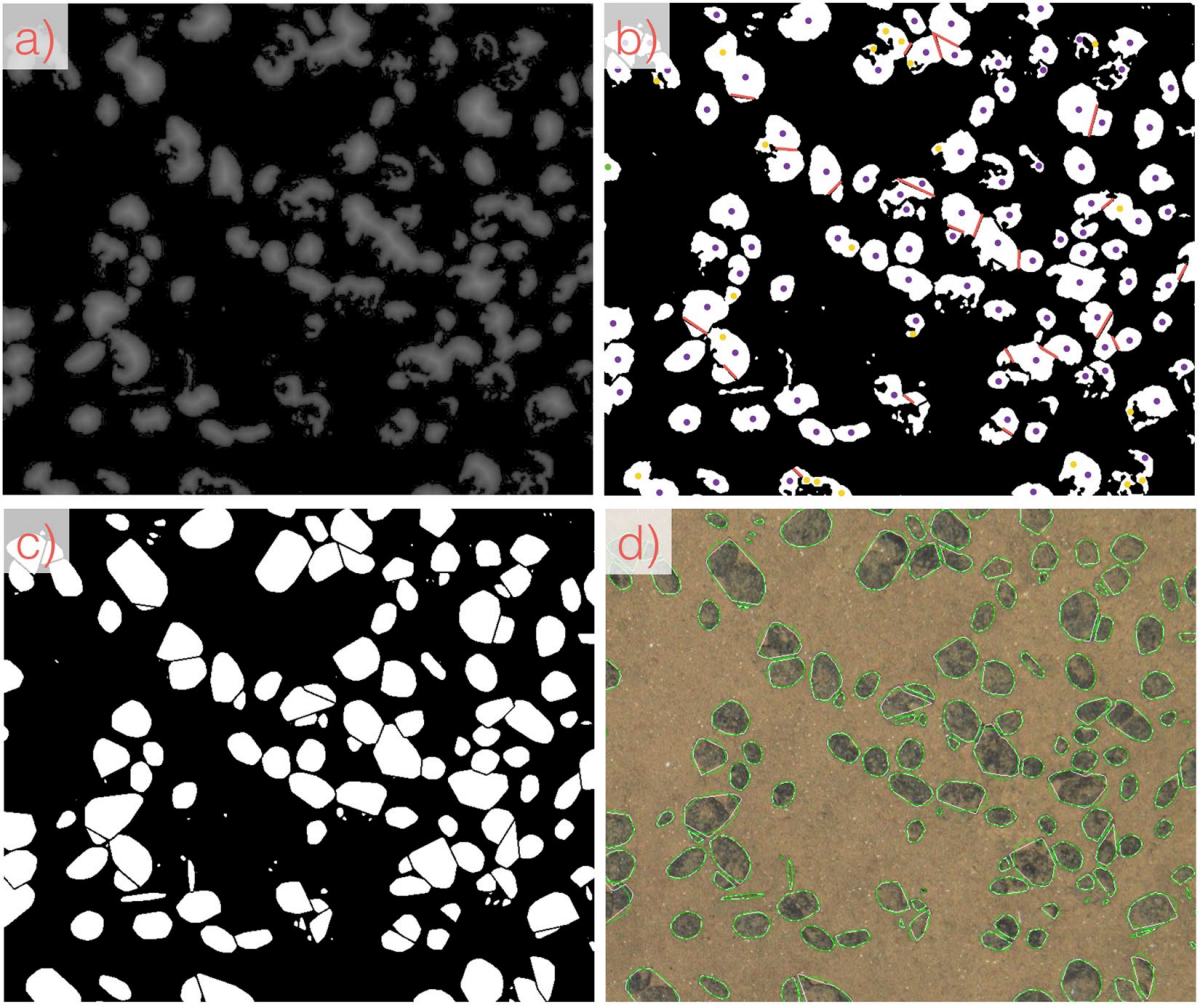
Workflow of the nodule delineation steps in the second phase of *CoMoNoD*. Again the zoom-in section is shown (see Fig. 2). (**a**) Distance image ${D}_{i}$ computed from ${B}_{i}$ (see Fig. 3c)). (**b**) Peaks within the distance image constitute nodule candidate centroids (yellow markers). These are filtered to determine the regional maximum within a neighbourhood (purple markers - all overlaying further yellow markers). Shape bottlenecks between adjacent nodules are used to separate connected nodule candidates (red lines). (**c**) each nodule candidate blob is delineated by its convex hull. (**d**) Convex hulls from c) are fit by an ellipsoid and shown as an overlay on top of the original image (green nodule delineations). The size of these delineations can be measured in cm^2^ and provides the basis for statistical assessments of PMN abundance.

The proposed *CoMoNoD* method consists of two phases: 1) contrast maximisation (see Figs 3 and 4) and 2) nodule delineation (see Fig. 5). The core heuristic - assuming that PMNs are elliptical, mostly convex objects - is exploited in both phases. All image processing is conducted using the OpenCV C++ library and GPU acceleration is used where applicable.

### Contrast maximisation

Each image ${I}_{i}$ of an image set $I$ is first filtered with a 3 × 3 px median filter to remove sensor noise and small artefacts. Artefacts can be caused by floating particles like marine snow or shell fragments on the seafloor. The images are then scaled with a scale factor ${f}_{i}={A}^{\ast }/{A}_{i}$ where ${A}_{i}$ is the footprint of image ${I}_{i}$ in m^2^. ${A}^{\ast }$ is the median image footprint in the image set $I$. Cubic interpolation is used while scaling the images. This is the most accurate interpolation method in the GPU-accelerated part of OpenCV. Scaling leads to a uniform px/cm ratio within the scaled image set.

**Figure 6:**
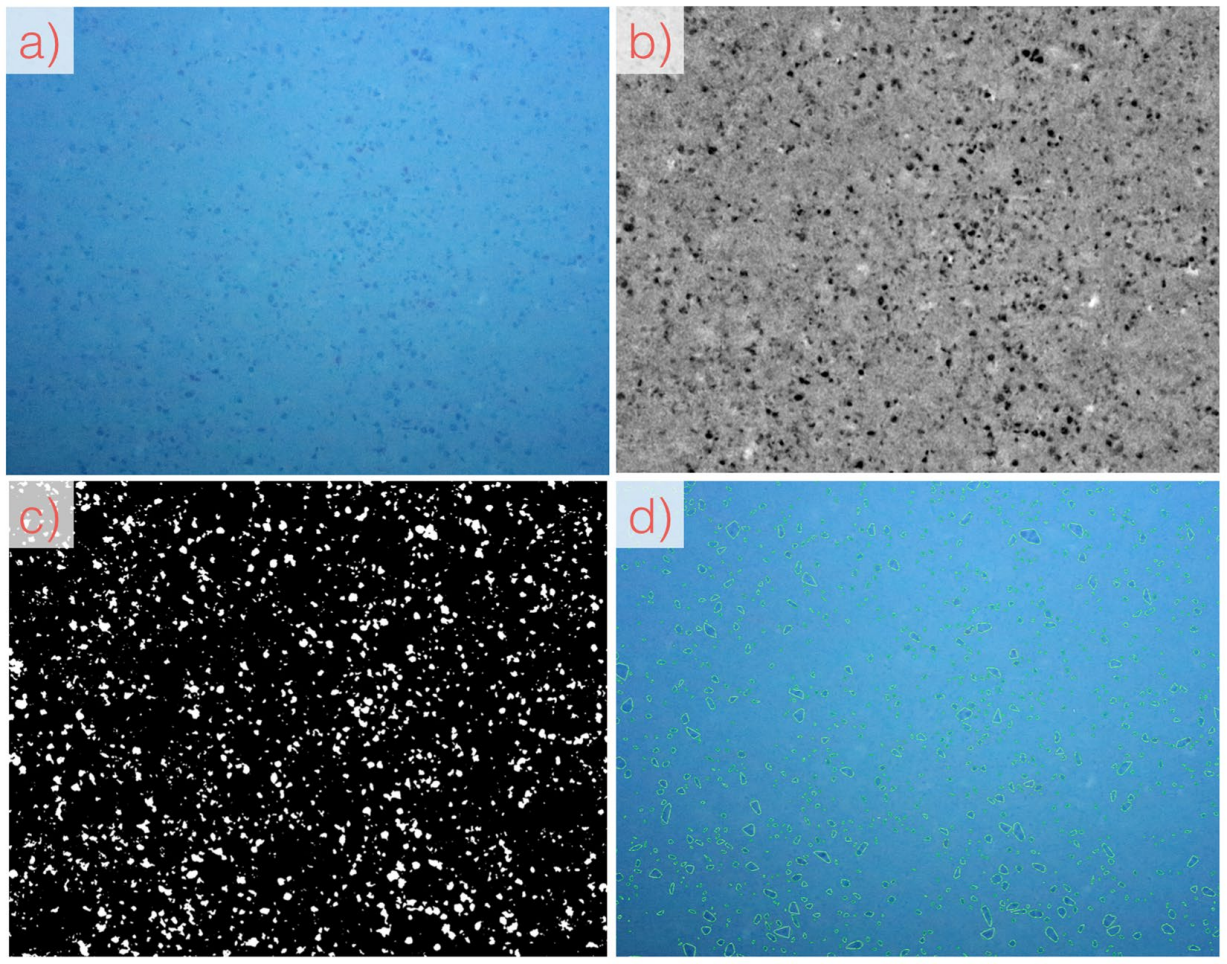
Processing workflow for ${I}^{\mathrm{(1)}}$. Shown are the zoom-ins to the original images (see Fig. 2). (**a**) Source image, (**b**) *fSpice* result, (**c**) binary image, (**d**) nodule delineation.

Scaled images are smoothed by a Gaussian filter of 3 × 3 px size. Afterwards, they are colour corrected using the *fSpice* algorithm (see Figs 3b) and 6(b))^11^. This data-driven colour correction method removes an illumination cone from individual images and equalises the colour histograms of all images within an image set. The algorithm was developed to reconstruct the natural appearance of the seafloor. Here, *fSpice* is applied in a way which increases the contrast between nodules and the sediment. The colour corrected images are then converted to 8 bit grey-scale.

Each grey-scale image is transformed to a binary image ${B}_{i}$ to maximise the contrast. An image-specific threshold ${t}_{i}$ is used for binarisation (${t}_{i}\in \mathrm{[0..255]}$, see Fig. 3c)). To determine ${t}_{i}$, first a grey level co-occurrence matrix ${G}^{(i)}$ is computed considering the four Moore-neighbouring pixels in 1 px distance. At this point the compactness heuristic is exploited: few colour co-occurrences of pixels below ${t}_{i}$ (likely PMNs - i.e. ${\omega }_{0}$) and above ${t}_{i}$ (likely background - i.e. ${\omega }_{1}$) are targeted. Hence for each ${t}_{i}$, a compactness value $\gamma ({t}_{i})$ is computed:

1 $$\gamma ({t}_{i})=\frac{\sum _{a\mathrm{=0}}^{t}\sum _{b\mathrm{=0}}^{t}{G}_{a,b}^{(i)}+\sum _{a=t+1}^{255}\sum _{b=t+1}^{255}{G}_{a,b}^{(i)}}{\sum _{a\mathrm{=0}}^{255}\sum _{b\mathrm{=0}}^{255}{G}_{a,b}^{(i)}}\in \mathrm{[0..1]}$$

This $\gamma ({t}_{i})$ is maximised for $\gamma \mathrm{(0)}=\gamma \mathrm{(255)}=1$.

Next, the first derivative of $\gamma ({t}_{i})$ is computed and its peak position ${p}_{i}$ determined:

2 $${p}_{i}=\mathop{{\rm{argmax}}}\limits_{a}\gamma ^{\prime} (a\mathrm{).}$$

The threshold ${t}_{i}$ is selected as:

3 $${t}_{i}=\gamma ^{\prime} \mathop{{\rm{argmax}}}\limits_{b < {p}_{i}}\gamma ^{\prime} (b)$$

with:

4 $$\gamma ^{\prime} (b) < \gamma ^{\prime} ({p}_{i})\cdot {\theta }_{\gamma }$$

where ${\theta }_{\gamma }$ is one of the *CoMoNoD* input parameters.

The concept for selecting the threshold ${t}_{i}$ like this is as follows. The compactness $\gamma ({p}_{i})$ decreases when the binary image ${B}_{i}$ becomes noisy. This is the case when background pixels are erroneously added to the PMN class ${\omega }_{0}$ (see Figs 3d) and 4). It will happen when ${t}_{i}$ is chosen too high. The maximum compactness change $\gamma ^{\prime} ({p}_{i})$ thus serves as the upper limit for the threshold value ${t}_{i}$.

### Nodule delineation

After contrast maximisation, the binary image ${B}_{i}$ is subject to multiple blob detection, splitting and fusion steps. These steps delineate individual nodules in the second phase of *CoMoNoD* (see Fig. 5).

First, the distance image ${D}_{i}$ is computed, which has the same size as ${B}_{i}$. Each pixel value in ${D}_{i}$ encodes the shortest distance to a pixel in ${B}_{i}$ assigned to class ${\omega }_{1}$ (see Fig. 5a)).

Local maxima are determined in ${D}_{i}$. Maxima are pixels that exceed their Moore-neighbouring pixels. These local maxima constitute the initial nodule candidate centroids. Neighbouring local maxima are filtered and only the highest peak is retained within a $5\ast {\theta }_{r}$ px radius (see Fig. 5b)). ${\theta }_{r}$ is another input parameter for *CoMoNoD*. It represents the minimum nodule radius in pixels and depends on image resolution.

Each pixel in ${B}_{i}$, which is set to ${\omega }_{0}$, needs to be assigned to one of these peaks. Here it is again assumed that PMNs are compact objects of elliptical shape. The pixels set to ${\omega }_{0}$ form connected pixel clusters or *blobs*.

Bottlenecks in the blob contours are evaluated to separate adjacent PMNs in the binary image (see Fig. 5b) - red lines). Each blob is iteratively split up to virtual blobs to find the optimal separation of the peaks within the blob. The iterative splitting is discontinued when each peak is contained in its own blob (see Fig. 5b)).

All pixel blobs are fused with their largest neighbour when they are smaller than $\pi \ast {\theta }_{r}^{2}$ pixels. To be fused the neighbour has to be closer than $2\ast {\theta }_{r}$ pixels distance. Fusion avoids over-segmentation. Small blobs are discarded if no such neighbour exists.

Nodule candidate blobs are delineated by their convex hull to account for gaps between PMNs. Gaps can be caused by sediment coverage or epifauna (see Fig. 5c)). Each convex hull is finally fit with an ellipsoid (see Fig. 5d)). The size of these ellipsoids provides individual nodule sizes in cm^2^ from which descriptive nodule statistics can be computed. The main axes of the ellipse also provide measurements of two of the nodule axes.

### Nodule statistics

The number of PMNs in an image is denoted by ${N}_{i}$. Each PMN nodule ${n}_{i,j},j=0,\,\mathrm{..,}\,{N}_{i}$ in an image ${I}_{i}$ is described by a size value ${s}_{i,j}$ in cm^2^. Several PMN abundance statistics can be computed from these ${s}_{i,j}$. The most straightforward are the number of PMNs per square meter (${{\rm{\Phi }}}_{i}^{N}={N}_{i}/{A}_{i}$ m^−2^) and the percentage coverage of the seafloor with PMNs (${{\rm{\Phi }}}_{i}^{c}=\sum _{j\mathrm{=0}}^{{N}_{i}}{s}_{i,j}/{A}_{i}$). To represent the nodule size distribution in an image, *CoMoNoD* additionally provides seven characteristic nodule size values:

5 $${{\rm{\Phi }}}_{i,\alpha }^{s},\alpha \in \mathrm{[0.01,0.10,0.25,0.5,0.75,0.90,0.99]}$$

These are computed by first sorting the ${s}_{i,j}$ by increasing value. Then:

6 $${{\rm{\Phi }}}_{i,\alpha }^{s}={s}_{i,{k}_{\alpha }}$$

with:

7 $${k}_{\alpha }=\mathop{{\rm{argmax}}}\limits_{k}\sum _{l=0}^{k < {N}_{i}}{s}_{i,l} < \alpha \cdot {{\rm{\Phi }}}_{i}^{c}\cdot {A}_{i}$$

The interpretation of ${{\rm{\Phi }}}_{i,\alpha }^{s}$ is that a fraction of $\alpha$ of ${{\rm{\Phi }}}_{i}^{c}$ in image ${I}_{i}$ is owing to nodules smaller than ${{\rm{\Phi }}}_{i,\alpha }^{s}$ (e.g. ${{\rm{\Phi }}}_{i\mathrm{,0.5}}^{s}$: half of the nodule coverage in ${I}_{i}$ is created by nodules smaller than ${{\rm{\Phi }}}_{i\mathrm{,0.5}}^{s}$ cm^2^). The ${{\rm{\Phi }}}_{i,\alpha }^{s}$ are thus values along the accumulation curve of the nodule size distribution.

Common geological particle size descriptors can be computed from the ${{\rm{\Phi }}}_{i,\alpha }^{s}$ ^31^. An example are the sorting value: $0.5\cdot ({{\rm{\Phi }}}_{i\mathrm{,0.75}}^{s}-{{\rm{\Phi }}}_{i\mathrm{,0.25}}^{s})$ and the skewness: ${{\rm{\Phi }}}_{i\mathrm{,0.75}}^{s}+{{\rm{\Phi }}}_{i\mathrm{,0.25}}^{s}-2\cdot {{\rm{\Phi }}}_{i\mathrm{,0.5}}^{s}$ as defined by Trask^32^.

## Results

Figure 6 shows an example of the delineation result for ${I}^{\mathrm{(1)}}$ (in d)). It also shows intermediate results of the *fSpice* pre-processing and the binarization step. An example delineation is shown in Fig. 5d) for ${I}^{\mathrm{(2)}}$.

**Figure 7:**
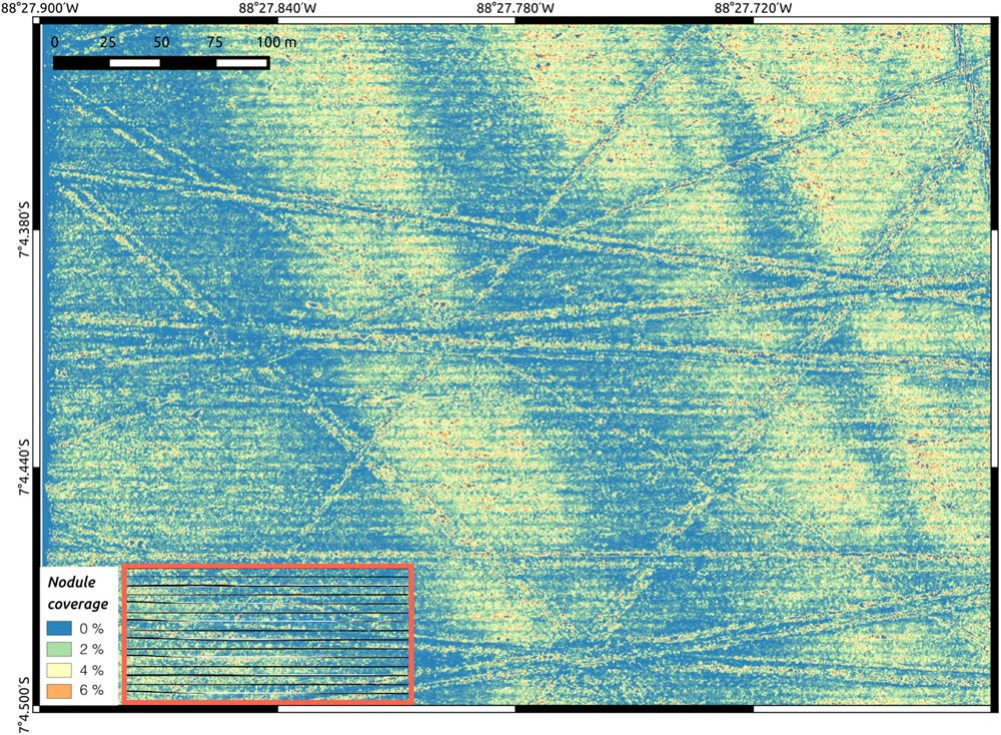
Nodule abundance map showing nodule coverage in percent. 34,200 images contributed to this map. It was computed in about 12 h. The map represents an area of ca. 500 × 400 m^2^ size. It lies in the DISCOL experimental area in the Peru Basin, Pacific Ocean. Narrow linear structures, criss-crossing the area, are anthropogenic plough-marks (8 m wide) from a mining simulation conducted in 1989^28^. The larger scale (ca. 100 m wide), east-west facing wave pattern correlates with the seafloor micro-bathymetry. The striped horizontal pattern follows the AUV dive trajectory (see black lines in the inset in the lower left corner). Eighty horizontal AUV lines were conducted at a spacing of 5 m. The horizontal pattern is likely caused as an artefact of the max-pooling value selection for overlapping images.

By applying *CoMoNoD* to all 34,200 images in ${I}^{\mathrm{(1)}}$, nodule abundance was assessed within a contiguous seafloor area of ca. 500 × 400 m^2^. Nodule abundance was quantised to a 0.25 × 0.25 m^2^ resolution grid to render the map (see Fig. 7). Therefore, each image was subdivided into 25 × 25 cm^2^ tiles. *CoMoNoD*’s descriptive statistics were then computed for each of these grid tiles.

**Figure 8:**
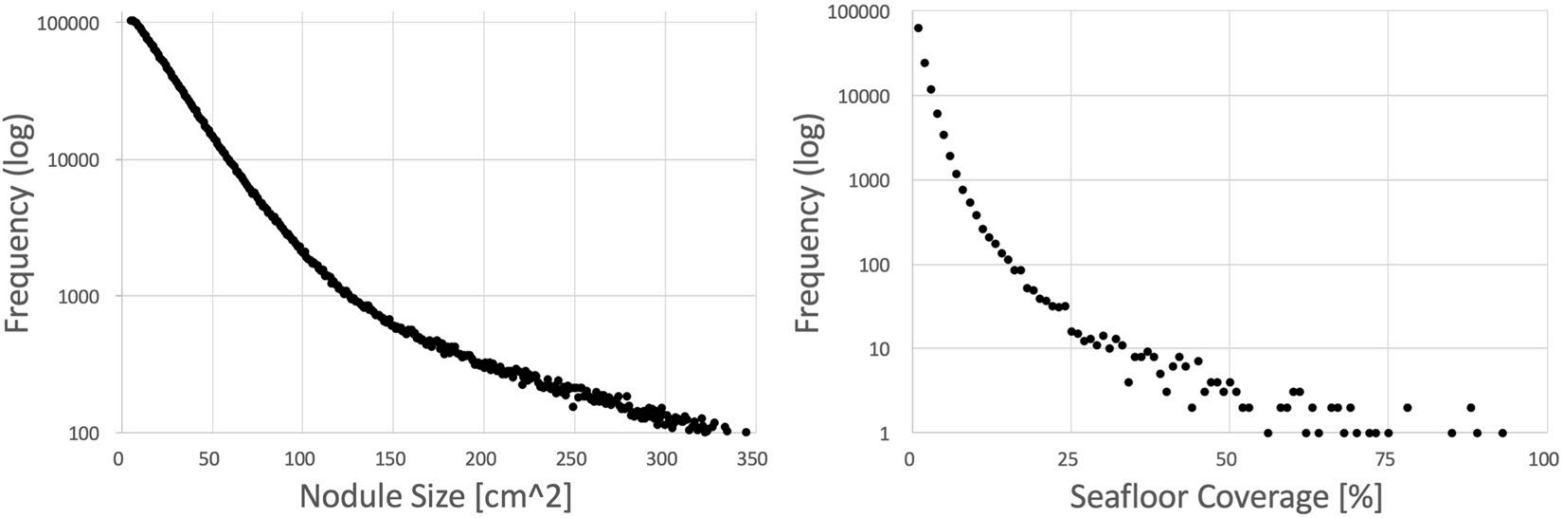
Nodule size distribution (left) and coverage distribution (right) for ${I}^{\mathrm{(1)}}$.

The average nodule number per square meter for the entire area is ${{\rm{\Phi }}}^{N}\mathrm{=2.7}$ m^2^, the average seafloor coverage is ${{\rm{\Phi }}}^{c}\mathrm{=1.7 \% }$ and the median nodule size is ${{\rm{\Phi }}}_{0.5}^{s}\mathrm{=48.6}$ cm^2^ (corresponding to a radius of 3.9 cm). Figure 8 shows the nodule size distribution and the coverage distribution.

More than 2.6 million nodules were delineated in the surveyed area. They cover a total area of 8.8 thousand square meters. The nodule volume can be estimated by assuming that the nodules are discoidal rotational ellipsoids. They would form a contiguous manganese nodule cube of 6.6 meter edge length. Assuming a metal composition as reported in the literature (Cobalt: 0.26%, Nickel: 1.2%, Copper: 1%)^33^, contained metal amounts can be deduced. The nodule cube would have an estimated worth of ca. 1 million EUR at current market prices (Cobalt: 69 kEUR/t, Nickel: 12 kEUR/t, Copper: 7.5 kEUR/t). These values are rough estimates, they do not consider the mining costs and completely neglect the value of the destroyed habitat and fauna.

Coverage values provided by *CoMoNoD* are comparable to results created by two other methods (*PCCA* ^24^ and *ES4C* ^23^) showing an agreement in the coverage estimates of ca. 92%.

Images in ${I}^{\mathrm{(2)}}$ were acquired along linear transect lines and no contiguous map can be rendered. The nodule statistics for all 88,630 images are ${{\rm{\Phi }}}^{N}=111.3$ m^−2^, ${{\rm{\Phi }}}^{c}=\mathrm{6.2 \% }$ and ${{\rm{\Phi }}}_{0.5}^{s}=10.4$ cm^2^ (corresponding to a radius of 1.8 cm).

Further nodule statistics are being computed for the remainder of the image set which ${I}^{\mathrm{(1)}}$ belongs to (cruises SO239 and SO242-1 - see above). Results will be presented in a follow-up publication.

### Runtime

*CoMoNoD* was implemented and tested on a high-end desktop computer. It was equipped with a GeForce GTX 980 Ti GPU, Intel Xeon E5-1650 CPU and 64GB RAM. An average runtime of ca. 0.1 s/MPix was observed. Computational complexity of the contrast maximisation is linear in the pixel size. Complexity of the nodule delineation steps is quadratic in the number of nodule segments. Thus images showing more nodules will lead to a longer runtime.

Images in ${I}^{\mathrm{(1)}}$ are ca. 20 MPix in size and the runtime for the entire data set was about 19 hours. Images in ${I}^{\mathrm{(2)}}$ are ca. 5 MPix in size, resulting in a runtime for the entire data set of about 12 hours. Table 1 shows average runtimes for image set ${I}^{\mathrm{(1)}}$.

**Table 1:** Runtimes for different nodule detection algorithms. *CoMoNoD* operates without a training phase and has the fastest application time per image. *PCCA* stands for *Pixel-classification by cluster annotation*. This method has been improved by a software company to create *Rapid PCCA*. *ES4C* stands for *Evolutionary tuned Segmentation using Cluster Co-occurrence and a Convexity Criterion*. It first uses the same PR methods as *PCCA* in the training phase. An additional optimisation step is conducted in the training phase of *ES4C*. It allows omitting the manual cluster annotation required in *PCCA*. Training of *PCCA*, *Rapid PCCA* and *ES4C* is conducted on a data subset corresponding to ca. 5 images.

Method	Training [min / MPix]	Training for ${{\boldsymbol{I}}}^{{\bf{(1)}}}$ [h]	Application [s / MPix]	Application to ${{\boldsymbol{I}}}^{{\bf{(1)}}}$ [h]
*CoMoNoD*	—	—	0.1	19
*PCCA* ^24^	4.7	3.9	1.9	233
*Rapid PCCA* ^25^	4.7	3.9	0.2	25
*ES4C* ^23^	10.3	8.6	1.9	233

### Source Code

The source code for *CoMoNoD* is available from the GEOMAR OpenSource Git repository (https://git.geomar.de/open-source/comonod). Code in this repository is maintained to include future algorithmic updates. A snapshot of the code used for this publication has been archived in Pangaea^34^. Additional software required to run the algorithm is the OpenCV image processing library (available through http://opencv.org/) and the *fSpice* algorithm (https://git.geomar.de/open-source/oceancv).

## Discussion

So far, assessing PMN abundance variations was not possible over multiple spatial scales in near realtime. Measurements performed after physical sampling provide local abundance measurements. In case of box corers the sampled area represents 0.25 m^2^ of the seafloor. Patterns on scales larger than 0.5 m are missed. Ship-based hydro-acoustic data enables assessing nodule patterns at scales of several hundred square kilometres. Its beam-footprint of ca. 50 × 50 m^2^ oversees patterns on smaller scales.

Optical imaging provides a tool to capture PMN abundance variations over several scales. Automated nodule delineation allows to rapidly extract quantitative data from those images. Applied as a joined methodology, imaging and automated analysis can assess PMN abundance and abundance variations from centimetre to kilometre scale.

*CoMoNoD* is currently applied to image data sets of several hundred thousand photos acquired across the Pacific Ocean. This study will provide nodule abundance statistics for local, regional and ocean basin scale.

Statistically assessing the natural heterogeneity requires to select a sampling size for the seafloor area *quants*. In physical sampling this is restricted to one specific size (e.g. 0.25 m^2^ for box cores). For hydro-acoustics the minimum sampling size depends on the beam opening angle and the distance to the seafloor (meter scale for AUV-based data, 40-100 m for ship-based data). For image-based methods, this area *quant* can be selected. Its size will affect the derived descriptive statistics. When choosing a very small area quant (e.g. 10 × 10 cm^2^) it will be rare to detect nodules. When a nodule does occur within the area, the coverage can reach 100%. Similarly, local nodule abundance heterogeneity might be occluded when the area is chosen too large (e.g. 100 × 100 m^2^). The summative character of the ${{\rm{\Phi }}}_{i}$ will then average out those variations. A study on assessing nodule abundance heterogeneity on various scales is in preparation and will provide suggestions for sampling size selection.

Abundance maps like Fig. 7 provide a subjective impression of the quality of the nodule delineation algorithm. Ground truth information on nodule abundance would be needed to verify the detection accuracy. As manual annotations are lacking for nodule image and as the quality of manual annotations is disputable, robust verification is currently impossible. Ground truth data from physical sampling could be linked with our image analysis method, yet the natural abundance heterogeneity and the limited accuracy of underwater navigation currently prohibit such an assessment. No data set exists where an image has been taken prior physical sampling to assure a one-to-one comparison of image-derived and ground truth data. Video material of TV-guided box cores is of too low quality to be analysed quantitatively.

The contrast maximisation step provides good binary images in all cases. Sediment coverage creates holes in the third example. The nodule delineation succeeds for the first two examples. Percent coverage could be measured in the latter two only.

To overcome these shortcomings, an imaging survey should be conducted prior to the physical seafloor sampling. This would allow assessment of the visual baseline of the undisturbed seafloor. The physical sampling afterwards would provide precise ground truth measurements of individual nodules. Accuracy of underwater navigation is far from the required accuracy of few tens of centimetres for direct comparison. To overcome ambiguities sampling sites need to be distant enough from each other ($>$50 m). This will allow identification of each sampling location at the seafloor based on underwater navigational data. A second imaging survey over the same area would then determine the exact positions of the sampling impact sites using visual navigation.

**Figure 9:**
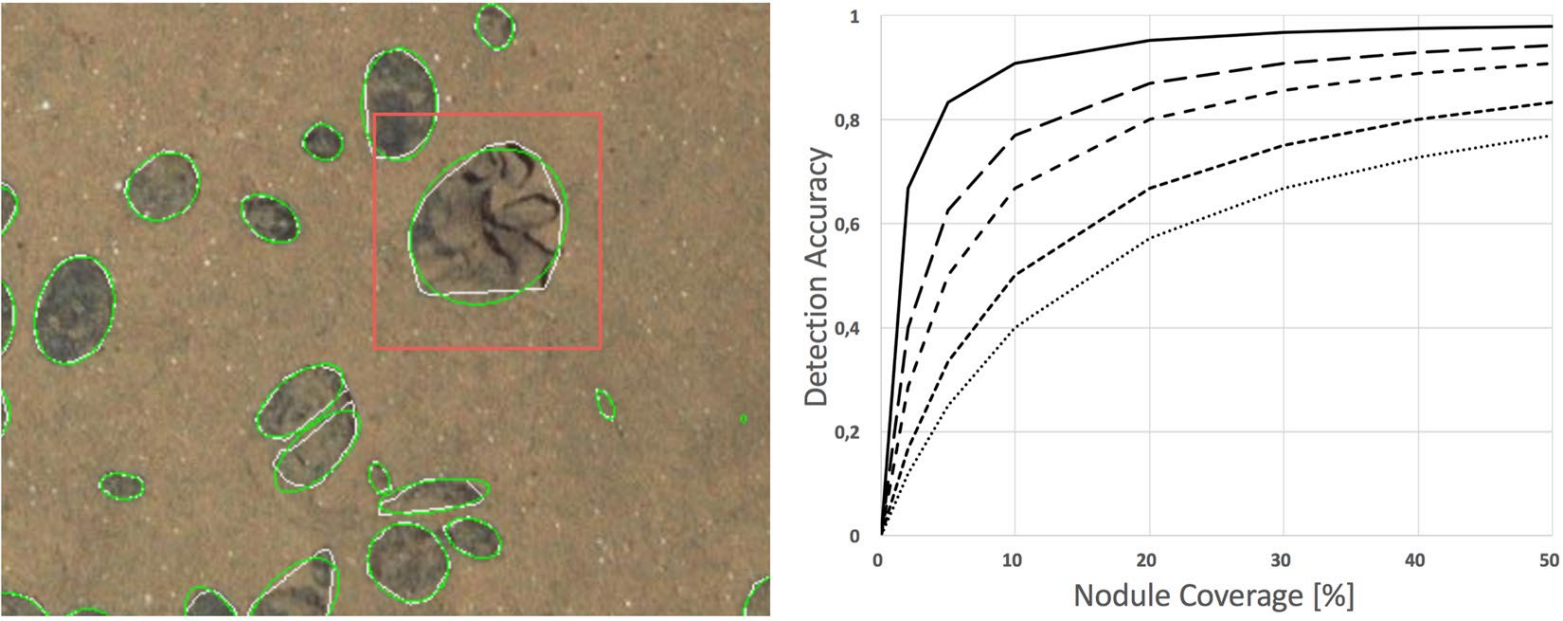
Zoom-in on a *Xenophyophore* that has been misclassified as poly-metallic nodule (left). Detection accuracy per observed nodule coverage (in %) for different fauna coverage values (right). The curves represent different pixel coverage by fauna (solid: 1%, long-dash: 3%, medium-dash: 5%, short-dash: 10%, dotted: 15%). *CoMoNoD* should be accompanied by other detection methods when dark fauna represents more than 10% of the pixels. Bright fauna will be assigned to the sediment class and will not skew the nodule statistics. For ${I}^{\mathrm{(1)}}$ and ${I}^{\mathrm{(2)}}$ dark fauna contributes to less than 3% of the pixels.

Frequently occurring fauna can become a challenge for *CoMoNoD*. Under such conditions, the assumption that these objects do not contribute to the nodule class does not hold any more. An example of a misinterpretation is shown in Fig. 9. There, a *Xenophyophore* has been erroneously detected as a PMN, contributing an additive error to the coverage and nodule count measures and likely affects ${{\rm{\Phi }}}_{i}^{s}$ as well. Interestingly it has been shown, that *CoMoNoD* can also be used to detect specific fauna by selecting images with outliers for ${{\rm{\Phi }}}_{i\mathrm{,0.99}}^{s}$. These outliers can be an indicator for large *Holothurians*. However, most complex objects are characterised by more complex visual features. Those objects require pattern recognition systems that are more sophisticated then *CoMoNoD*. Deep sea fauna can be colourful, complex-shaped or textured. Even nodules can present rich morphologies and colour features when imaged at high resolution. In those cases *CoMoNoD* can be challenged as well (see below).

As binary segmentation and delineating convex objects are also topics elsewhere, *CoMoNoD* could be applied in those contexts as well. Potential fields are microscopy (e.g. for blood cells) and remote sensing (e.g. for permafrost thaw craters).

**Figure 10:**
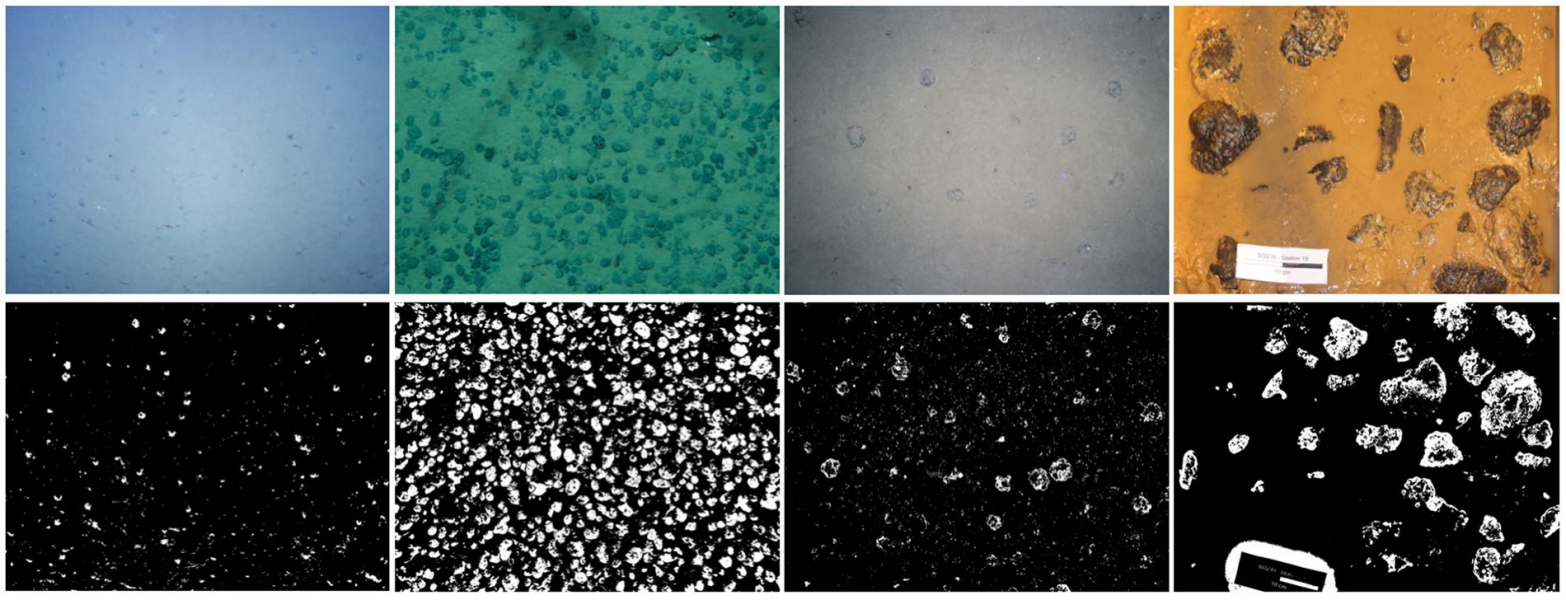
Examples showing *CoMoNoD* results for other imagery. From left to right: image acquired by *AUV Abyss* from lower altitude (resolution 320 Pix/m); image acquired by a towed camera by the Federal Institute for Geosciences and Natural Resources (1890 Pix/m); image acquired by a towed camera by the Alfred-Wegener Institute Helmholtz-Center for Polar and Ocean Research (1900 Pix/m)^35^; image acquired on deck by an SLR camera (8492 Pix/m).

*CoMoNoD* was applied to further nodule image data sets (see Fig. 10).

These were obtained by different camera systems mounted on different platforms. Illumination was different in terms of light sources and direction. The data sets are mostly *in-situ* imagery like ${I}^{\mathrm{(1)}}$ and ${I}^{\mathrm{(2)}}$. One data set shows *ex-situ* images of box core samplings acquired on deck of a research vessel. For all of these data sets, appropriate settings for ${\theta }_{\gamma }$ provided binary images with maximised contrast between nodules and the sediment background.

For the two image data sets with the highest pixel resolution, and thus highest pixel number per nodule, no appropriate nodule delineation could be achieved. This is likely because the high resolution makes the internal heterogeneity of the nodule itself visible and detectable by *CoMoNoD*. In such cases, *CoMoNoD* could be extended by a fallback system using surrounding pixel’s colour information (as described above). This would derive nodule outlines more robustly when the initial delineation does not provide satisfying results. Further studies are needed to determine the limits of the delineation process.

*CoMoNoD* is governed by the parameter ${\theta }_{\gamma }$. An intelligent parameter guessing system could be implemented that would again use the nodule convexity-heuristic. This system would compute delineations for various settings of ${\theta }_{\gamma }$ and ${\theta }_{r}$ in a brute-force manner. The most promising settings could then be determined by picking the one that produces mostly convex pixel blobs. It would also be possible to analyse multiple images with one parameter setting and assess the resulting nodule statistics as a quality criterion for the chosen parameters.

In the current implementation ${t}_{i}$ is estimated for each image. An improved *CoMoNoD* could use the estimate of the previous image to reduce the parameter search space for the current image. When images are acquired in quick succession (e.g. at 1 Hz) it can be assumed that they are rather similar. The nodule distribution, image illumination and seafloor distance should remain comparable and hence ${t}_{i}$ should be similar.

The second phase of *CoMoNoD* merges pixel blobs where nodules were cut into multiple segments. It also breaks up pixel blobs where nodules are connected to each other in ${B}_{i}$. These steps can fail when large epifauna occurs on a nodule or when a sediment cover occludes the visibility on the nodules. Both steps are steered by the compactness heuristic and ${\theta }_{r}$. Using aerial feature descriptors, further information about pixel neighbourhoods could be included in the nodule fusion and decision process. This additional information would come at an additional computational cost. Further intelligence could be added to *CoMoNoD* by making the initial median filter adaptive, i.e. using the median only when the intensity change exceeds a threshold. These improvements were purposely neglected for *CoMoNoD* to maintain its rapid data analysis capability.

For new users of the algorithm it is easy to explore the influence of different settings for ${\theta }_{\gamma }$ and ${\theta }_{r}$. A graphical user interface with two sliders would enable users to rapidly create nodule delineations for visual inspection. No feature computation, data normalisation or model training is necessary. *CoMoNoD* can directly be applied to single images. When promising settings have been determined, these can then be applied to the remainder of the data set in an automated way.

## Conclusion

The proposed *CoMoNoD* algorithm segments poly-metallic nodules from the sediment background by delineating individual nodules and extracting quantitative abundance data from optical seafloor imagery. It links to the traditional methods for nodule abundance assessment that use physical sampling and hydro-acoustic mapping. *CoMoNoD* is governed by only few simple parameters compared to other pattern-recognition-based nodule detection methods. Annotation is not required. Its rapid execution time enables quantitative mapping of nodule distributions during research cruises. *CoMoNoD* quantifies nodule abundance and abundance variations from square-centimetre to square-kilometre scale.
